# Angiotensin II type 2 receptor signalling as a pain target: Bench, bedside and back-translation

**DOI:** 10.1016/j.coph.2023.102415

**Published:** 2023-12-01

**Authors:** Andrew J. Shepherd, Andrew SC. Rice, Maree T. Smith

**Affiliations:** 1 The MD Anderson Pain Research Consortium and the Laboratories of Neuroimmunology, Department of Symptom Research, Division of Internal Medicine, The University of Texas MD Anderson Cancer Center, Houston, TX, USA; 2 Pain Research, Department of Surgery & Cancer, Imperial College London, London, UK; 3 School of Biomedical Sciences, The University of Queensland, St Lucia Campus, Brisbane, Queensland, Australia

## Abstract

Translating promising preclinical pain relief data for novel molecules from drug discovery to positive clinical trial outcomes is challenging. The angiotensin II type 2 (AT_2_) receptor is a clinically-validated target based upon positive proof-of-concept clinical trial data in patients with post-herpetic neuralgia. This trial was conducted because AT_2_ receptor antagonists evoked pain relief in rodent models of neuropathic pain. EMA401 was selected as the drug candidate based upon its suitable preclinical toxicity and safety profile and good pharmacokinetics. Herein, we provide an overview of the discovery, preclinical and clinical development of EMA401, for the alleviation of peripheral neuropathic pain.

## Introduction

The Global Burden of Disease study reveals that pain, along with mental health, is the single greatest contributor to disability worldwide [1-3]. Pharmacotherapy for neuropathic pain is succinctly summarized by the Special Interest Group on Neuropathic Pain (NeuPSIG) guidelines [4]. However, pain is often inadequately relieved by these agents and/or they cause dose-limiting side-effects [5]. For opioid analgesics, addiction liability is a major problem that has led to the ‘opioid crisis’ particularly in the United States of America [6]. The large unmet medical need of poorly relieved chronic pain has spurred a huge global research effort aimed at unravelling the complex pathobiology of chronic pain. To date, 430 mouse genes [7] and >90 human genes are implicated in the pathophysiology of chronic pain but precisely how these genes and their encoded targets combine to produce individual chronic pain conditions, is unclear (reviewed in Ref. [8]). Additionally, the ability of existing animal model-based approaches to deliver new analgesic drugs has largely proven to be limited [9]. In the following sections of this review, we address preclinical and clinical research on angiotensin II type 2 (AT_2_) receptor antagonists for the relief of chronic pain and in particular, peripheral neuropathic (nerve) pain.

## Small molecule AT_2_ receptor antagonists in rodent pain models

Research initiated in 2003 by Professor Maree Smith, used two rodent models of peripheral neuropathic pain to show that multiple small molecule AT_2_ receptor antagonists evoked dose-dependent reduction in hind-paw hypersensitivity that was abolished in AT_2_ receptor knockout (KO) mice (Table 1, reviewed in Ref. [8]). In subsequent studies, including replication work in an independent laboratory, these findings were extended to show that small molecule AT_2_ receptor antagonists alleviated hind-paw hypersensitivity in multiple rodent models (Table 1; reviewed in Ref. [8]).

More recently, AT_2_ receptor antagonist-evoked pain relief was shown in a mouse model of gout induced by intraarticular (i.a.) injection of monosodium urate crystals (100 μg/joint) and characterised by mechanical hypersensitivity, spontaneous and cold nociception, and oedema in the ankle joint [10]. Pain and inflammation were reduced significantly by i.a. injection of the small molecule AT_2_ receptor antagonist, PD123319 (10 pmol/joint), or by pre-treatment with oral PD123319 (1 mg/kg) in mice [10]. Confirming the AT_2_ receptor as the target, mechanical hypersensitivity did not develop in the ipsilateral hindpaws of mutant *Agtr2*^tm1a^ mice that effectively had the AT_2_ receptor knocked out [10].

In other work by Shepherd and colleagues [11], a single intraplantar (i.pl.) injection of Ang II (10—300 pmol) in mice evoked a prolonged period (24 h) of mechanical hypersensitivity in the ipsilateral hindpaws that was attenuated by co-i.pl. injection of PD123319 (10 pmol) and abolished in AT_2_ receptor KO mice [11]. Importantly, i.pl. co-administration of the AT_1_ receptor antagonist, losartan with Ang II (100 pmol), did not attenuate i.pl. Ang II-evoked mechanical hypersensitivity in the ipsilateral hindpaws and i.pl. Ang II evoked mechanical hypersensitivity in the ipsilateral hindpaws of AT_1_ receptor KO mice [11]. As intrathecal PD123319 at 10 pmol did not attenuate i.pl. Ang II-evoked mechanical hypersensitivity in the ipsilateral hindpaws of mice, this indicated that i.pl. PD123319 evoked its antihyperalgesic effect via a peripheral mechanism [11].

## AT_2_ receptor crystal structure

**Figure 1:**
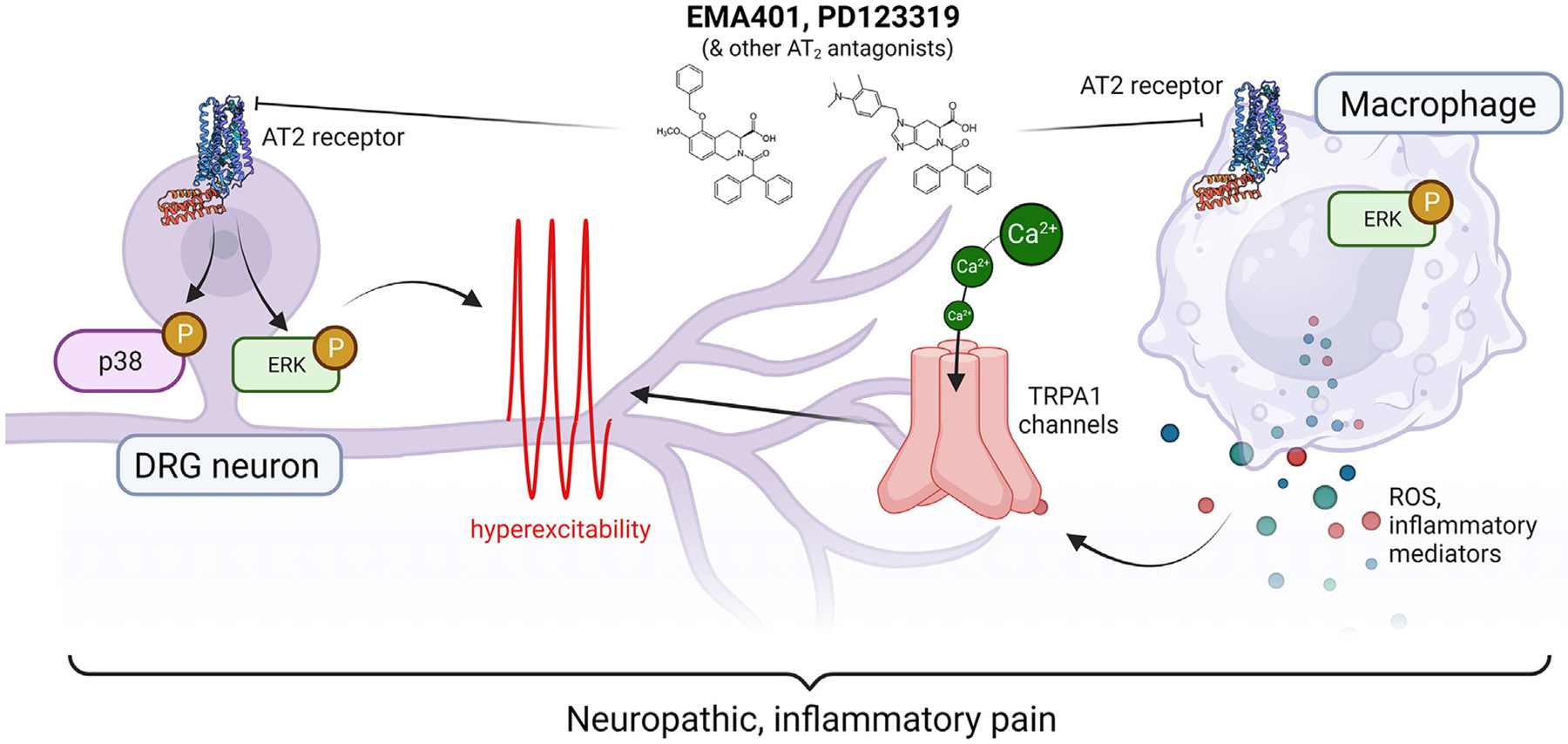
Proposed mechanism of action of AT_2_ receptor antagonists. EMA401, PD123319 and other antagonists of the AT_2_ receptor are thought to prevent the activation of ERK and p38 MAPK in sensory neurons. There is also evidence for inhibition of ERK activation via AT_2_ in macrophages. These signaling events reduce hypersensitivity in two ways, either by dampening neuronal excitability directly or, via suppression of macrophage-derived reactive oxygen species, attenuating hyperexcitability driven by the damage-sensing ion channel TRPA1.

For decades, the AT_2_ receptor was described as ‘enigmatic’ because neither Ang II nor an AT_2_ receptor agonist, induced G-protein or β-arrestin signal transduction despite the AT_2_ receptor being G-protein coupled (reviewed in Ref. [8]). The reason for this atypical AT_2_ receptor behaviour became apparent once its crystal structure was published in 2017 which showed that helix 8 of the AT_2_ receptor does not adopt the canonical position of G-protein coupled receptors (GPCRs) parallel to the membrane pointing outside of the TM7 bundle [12]. Instead, it interacts with the intracellular ends of transmembrane (TM) 3, TM5 and TM6 to impede engagement of classical GPCR effectors such as G-proteins and β-arrestin [12]. Subsequently, the AT_2_ receptor crystal structure bound to the AT_2_ receptor antagonist, EMA401, showed close similarity with previously determined structures of the AT_2_ receptor [13]. However, EMA401 had a different binding pose compared with other ligands [13]. Molecular docking showed that the lower binding affinity of PD123319 relative to EMA401 (Figure 1) at the AT_2_ receptor [14], was likely due to changing a benzene ring in EMA401 to an imidazole in PD123319 which reduced the stacking interaction with the guanidine group of R182 in ECL2 [13].

## Ang II signalling via the AT_2_ receptor activates p44/p42 mitogen activated protein kinase (MAPK)

Painful neuromas in humans and experimental neuromas in rats have high expression levels of pERK1/2 and pp38 MAPK which can phosphorylate multiple voltage-gated ion channels that are expressed by dorsal root ganglia (DRG) neurons and that are implicated in the pathobiology of neuropathic pain [9]. In the chronic constriction injury (CCI) of the sciatic nerve rat model of neuropathic pain [15] and in a rat model of mixed inflammatory and neuropathic pain with fully developed hindpaw hypersensitivity [16], there were augmented levels of pp38 MAPK and pERK1/2 in the ipsilateral lumbar DRGs that were attenuated to sham-levels at the time of peak effect of an AT_2_ receptor antagonist. These findings are aligned with *in vitro* work by Anand and colleagues who showed that exposure of cultured rat DRG neurons to Ang II and the AT_2_ receptor agonist, compound 21, increased pp38 MAPK and pERK formation that was attenuated by co-exposure of these DRG neurons to EMA401 [17] (Figure 1).

## Ang II signalling via the AT_2_ receptor and transactivation of TRPA1 in mice

As previously mentioned, i.pl. Ang II led to a dose-dependent increase in mechanical hypersensitivity in the ipsilateral hindpaws of wild-type mice that was absent in AT_2_ knockout mice. This pain hypersensitivity was associated with increased production of reactive oxygen species (ROS) at the site of injection, as revealed by *in vivo* imaging with a ROS-sensitive dye. This ROS production was attenuated by co-administration of PD123319 or the antioxidant N-acetylcysteine [11]. This suggested that a ROS-dependent mechanism could be responsible for Ang II/AT_2_-driven pain. Using the same i.pl. Ang II model in mice, deletion or blockade of the damage-sensing nociceptive ion channel TRPA1 (transient receptor potential, ankyrin family member 1) blocked Ang II-induced hypersensitivity in pain behaviour assays [18]. Observations in other pain models suggest that ROS-mediated activation of TRPA1 expressed by Schwann cells can also generate and sustain pain behaviours [19-21]. TRPA1 activation by ROS is thought to be mediated by membrane lipid peroxidation products modifying cysteine residues in mouse and human TRPA1 [22]. Consistent with these observations, heterologous expression of mutant TRPA1 constructs *in vitro* (which lack key modifiable cysteine residues) blocked the effect of Ang II-induced ROS on neuronal calcium flux [11] (Figure 1).

## Peripheral macrophages and the AT_2_ receptor

Our work [11] and that of others [23] demonstrated that painful neuropathy due to chemotherapy and diabetes in patients is associated with accumulation of macrophages in the skin, in close proximity to degenerating peripheral nerve terminals. Using transgenic mice and RT-PCR, we showed that macrophages in skin expressed the AT_2_ receptor [11], consistent with earlier reports that expression of AT_2_ and other components of the renin-angiotensin system were detectable in monocytes with expression increased as these cells differentiated into macrophages [24]. Since macrophages are prodigious ROS producers, it was hypothesized that the Ang II-induced ROS production in hindpaw skin that was associated with pain hypersensitivity via TRPA1 activation could be macrophage-driven. This is because acute chemogenetic ablation of macrophages was associated with loss of mechanical and cold hypersensitivity in nerve injury mice, and reconstitution of wild-type mice with AT_2_-KO donor bone marrow significantly attenuated nerve injury-induced hypersensitivity [25]. Additionally, *in vitro* exposure of peritoneal macrophages to Ang II resulted in AT_2_-dependent production of ROS that was absent in peritoneal macrophages derived from AT_2_-KO mice [11]. Furthermore, co-culture of AT_2_-expressing macrophages with sensory neurons was required to detect the Ang II-induced TRPA1 activation on sensory neurons [11] (Figure 1).

## Preclinical pharmacokinetics of small molecule AT_2_ receptor antagonists and *in vitro* metabolism of EMA401

Comparison of the preclinical pharmacokinetics of several small molecule AT_2_ receptor antagonists showed that EMA401 had favourable pharmacokinetics in the rat and hence it was chosen as the drug candidate. Specifically, the mean dose-normalized peak plasma concentration and systemic exposure of oral EMA401 in rats were 88.5 kg/mL and 300 h kg/mL, respectively [8]. Plasma clearance was 1.1 L/h/kg and the oral bioavailability was 33 % [8]. Interestingly, EMA401’s volume of distribution in rats was relatively low at 3.7 L/kg suggestive of a limited ability to cross the blood-brain-barrier, which was confirmed subsequently using ^14^C-labelled EMA401 and whole-body autoradiography in the rat to show an absence of ^14^C in the central nervous system [26,27]. Comparison of the *in vitro* metabolism of EMA401 in mouse, rat, dog, monkey and human hepatocytes, showed its metabolic stability was relatively low and that it underwent extensive glucuronidation [28]. In human hepatocytes *in vitro*, the predominant metabolic pathways were O-demethylation, acyl glucuronidation and O-debenzylation whereas in animal species O-glucuronidation and glutathione conjugation were also pronounced [28]. In human liver microsomes, the IC_50_ values for EMA401 inhibition of the metabolism of six probe substrates specific for individual cytochrome P450s (1A2/2C8/2C9/2C19/2D6/3A4) were >50/20/7.4/>50/>50/>50 showing that EMA401 has a medium inhibition of CYP2C9 [27].

## Preclinical toxicology and safety pharmacology studies

An investigational new drug (IND)-enabling toxicology (in rats and dogs) and safety pharmacology program of oral EMA401 completed by Spinifex Pharmaceuticals, showed that oral EMA401 was suitably safe to administer to healthy human volunteers in Phase 1 clinical trials as there were no significant toxicity effects in rats and dogs. Also, for hepatocytes incubated for 2 h with EMA401 for *in vitro* metabolism studies, there was no hepatocyte toxicity reported [28]. Unexpected hepatotoxicity was found in monkeys in a long-term (39-week) toxicity study that was conducted concurrently with Phase 2b clinical trials that resulted in the termination of these trials (see next section).

## Early phase clinical trials of EMA401

### Phase 1 clinical trials

The Phase 1 clinical trial program sponsored by Spinifex Pharmaceuticals comprised single ascending dose and multiple ascending dose arms in healthy volunteers as well as an assessment of the effect of food on the pharmacokinetics of oral EMA401. The Phase 1 trials were conducted in both healthy young and healthy older subjects as there is a higher prevalence of neuropathic pain in older patients. The maximum tolerated oral doses were 2000 mg (single dose) and 800 mg twice-daily [29]. Importantly, EMA401 had linear pharmacokinetics and an oral bioavailability of approximately 30 % in humans [29], mirroring that seen in rats [14].

### Phase 2a and 2b clinical trials of EMA401

To date, three double-blind, randomised, placebo controlled, clinical trials exploring the efficacy of AT_2_ receptor antagonists in chronic neuropathic pain have been reported. These data tend to support the hypothesis of AT_2_ receptor mediated analgesia in people living with postherpetic neuralgia or painful diabetic neuropathy, although further efficacy and safety data are required.

**Figure 2:**
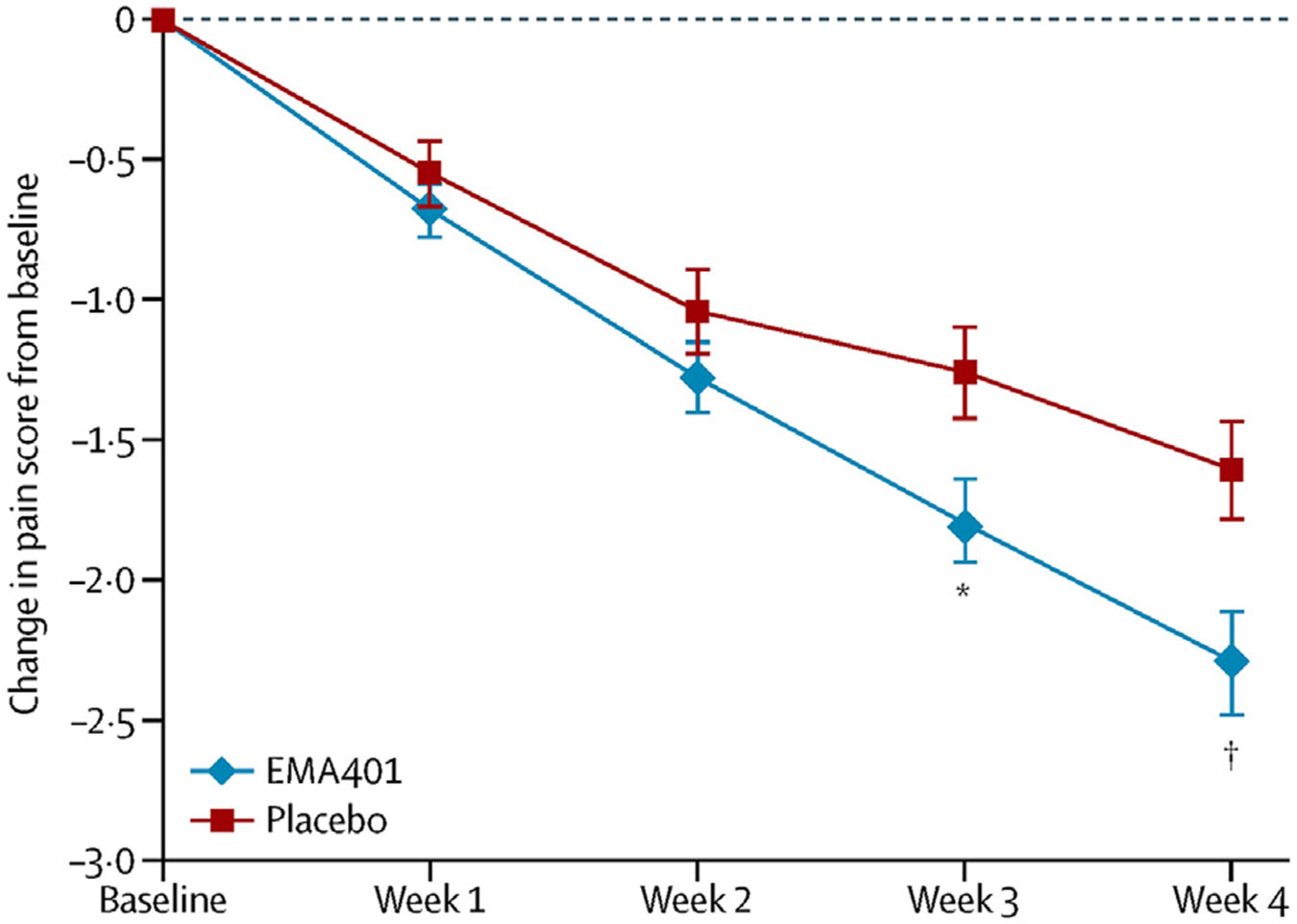
In a randomized, double-blind, parallel group, placebo-controlled, phase 2a clinical trial in patients with postherpetic neuralgia (PHN) of at least 6-month duration, twice-daily dosing with oral EMA401 (sodium salt) at 100 mg for 4 weeks evoked superior relief of PHN relative to placebo at the end of 28 days of treatment with significant differences between the 2 groups evident by day 21 of treatment. Reproduced from Ref. 30, with permission from Elsevier.

A phase 2 trial assessing EMA401 in people living with postherpetic neuralgia was conducted by Spinifex Pharmaceuticals [30]. Using a parallel group design, a single orally administered dose of EMA401 (100 mg b.i.d.) was compared to placebo over a 28-day treatment phase. Ninety-two participants were randomised (86 completed) to treatment with EMA401 and 91 participants to placebo (83 completed). Analysis of the primary efficacy variable (change in mean pain intensity between baseline and final dosing week) demonstrated superiority of EMA401 (mean reduction on 11-point intensity scale 2.29) over placebo (1.60) (Figure 2). With a Number Needed to Treat (50 % pain relief) of 6.7, EMA 401 100 mg b.i.d demonstrated efficacy comparable to existing neuropathic pain therapies. Treatment-emergent adverse events were reported by 3 % of EMA401 treated participants compared to 1 % of those randomised to placebo. No serious adverse events related to EMA401 were reported.

Following the acquisition of EMA401 by Novartis two phase 2b double blind, randomised, placebo controlled, multicentre trials were reported in a single publication [31]. The EMPADINE study compared a single dose of oral EMA401 (100 mg b.i.d.) to placebo in participants with painful diabetic polyneuropathy, whereas EMPHENE compared two doses of EMA401 (25 and 100 mg b.i.d.) to placebo in participants with post-herpetic neuralgia. A 12-week treatment period was evaluated. Both studies were terminated prematurely because of a preclinical primate toxicity finding of hepatotoxicity on long-term dosing. No similar signal has yet been reported in humans. Therefore, formal statistical analysis of the efficacy data was not appropriate, although the planned primary efficacy variable was a change in the weekly mean of the 24-h average pain score comparing baseline values to those in the final week of treatment (week 12). However, the reduction in numeric rating scale pain score was numerically in favour of EMA401 100 mg in both trials at treatment week 12. In EMPHENE a 300 mg dose was planned but not initiated, so the benefit of EMA401 at doses higher than 100 mg b.i.d. remains unknown.

A limitation of all three trials is that they were not designed to explore, and hence did not report, biomarker linked stratification of responses at the individual participant level. As people living with neuropathic pain represent a heterogeneous group this information is important when precision medicine approaches are contemplated. We suggest that future trials consider this approach. Stratification by sensory profile biomarkers is one such approach which has been advocated and the methods have been recently simplified to enable deployment in multicentre trials [32-34]. Based on the preclinical studies it would be reasonable to hypothesise that patients presenting with a sensory gain phenotype might be expected to respond to EMA401 [35].

## Conclusions

The AT_2_ receptor is a clinically-validated target for alleviating peripheral neuropathic pain, Although the clinical development of EMA401 was halted, second-generation small molecule AT_2_ receptor antagonists are in development for the relief of peripheral neuropathic pain, based upon the hypothesis that EMA401 hepatotoxicity in monkeys is molecule specific.

## Figures and Tables

**Table 1:** Selective, small molecule, AT_2_ receptor antagonists alleviate pain-like behavior in rodent models of neuropathic and chronic inflammatory pain.

Pain Type	Rodent model and pain-like behavioral endpoints	AT_2_R antagonist(s) evaluated and dosing regimen	ED_50_ (95%CI) (mg/kg)			Ref
			EMA200	EMA300	EMA400/EMA401 or analog	
Neuropathic	CCI of the sciatic nerve in the rat; mechanical allodynia	Single i.p. bolus doses; EMA200 1–10 mg/kg; EMA300 1–10 mg/kg; EMA400 0.003–0.03 mg/kg	3.22 (2.02–5.14)	0.78 (0.08–7.68)	0.013 (0.008–0.021)	[14]
		Single oral dose; EMA401 (30 mg/kg)	NA	NA	✓	[27]
		Single oral dose; Compound 15 (EMA401 analogue)	NA	NA	✓	
	ddC-induced ATN in the rat; mechanical allodynia (single doses); mechanical hyperalgesia (repeat doses) SNI of sciatic nerve in mice; mechanical and cold allodynia	Single i.p. bolus doses; EMA200 at 0.3–10 mg/kg	3.2 (1.43–7.0)	NA	NA	[36]
		Twice-daily i.p. doses; EMA300 at 1, 10 & 30 mg/kg for 3 days	NA	✓	NA	
		Single i.p. dose; EMA200 at 10 mg/kg	✓	NA	NA	[25]
		Single peri-sciatic dose of EMA200 (30 nmol)	✓	NA	NA	
		Single intrathecal dose of EMA200 (30 nmol)	Inactive	NA	NA	
	Acute paclitaxel neuropathic pain in mice; mechanical and cold allodynia	Single i.v. doses as prevention protocol; i.v. EMA200 at 20 mg/kg Oral EMA401 at 10 ng/kg	✓	NA	✓	[37]
Inflammatory	Unilateral i.pl. FCA-induced inflammatory pain in the rat; mechanical and thermal hyperalgesia	Single i.p. dose of EMA200 at 10 mg/kg on day 7; chronic i.p. dosing of EMA200 at 5 mg/kg/day for 7 consecutive days as a prevention protocol	✓ ✓	NA	NA	[38]
	Unilateral i.pl. FCA-induced inflammatory pain in the rat	Single i.p. dose of EMA200 at 10 mg/kg on day 2, day 4 or day 7	**X**			[11]
	FCA-induced monoarthritic pain in the rat knee joint	Single i.p. bolus doses of EMA300 and EMA400 at 0.1–10 mg/kg	NA	✓	✓	[39]
	FCA-induced vestibulodynia in the rat; peri-vaginal mechanical allodynia	Chronic i.p. doses of EMA200 at 5 mg/kg/day) for 7 consecutive days as a prevention protocol	✓	NA	NA	[40]
Inflammatory and neuropathic	Prostate cancer-induced bone pain in the rat; mechanical allodynia and thermal hyperalgesia in ipsilateral and contralateral hindpaws	Single i.v. doses of EMA200 at 0.3–10 mg/kg	0.8 (0.61–1.03) ipsi; 1.8 (1.2–2.8) contra; 3.9 (3.0–5.2 ipsi; 5.5 (3.9–7.7) contra	NA	NA	[16]
Acute pain	i.pl. or intrathecal Ang II in non-injured mice; mechanical allodynia	Single i.pl. dose of EMA200 (10 pmol) Single intrathecal dose of EMA200 (10 pmol)	✓ **X**	NA	NA	[11]
	i.a. monosodium urate (MSU) crystals (100 μg/ankle joint); mechanical allodynia, spontaneous nociception, cold nociception (acetone test) and joint inflammation	Single i.a. dose of EMA200 (10 pmol) co-injected with MSU	✓	NA	NA	[10]

Abbreviations. i.a. – intraarticular into the ankle joint; i.p. – intraperitoneal; i.v. – intravenous; CI – confidence interval; SNI = spared nerve injury; FCA – Freund’s complete adjuvant; ddC – dideoxycytidine; ATN – antiretroviral neuropathy; NA – not assessed; ipsi – ipsilateral; contra – contralateral.

## References

[1] Rice ASC, Smith BH, Blyth FM: Pain and the global burden of disease. Pain 2016, 157:791–796. 26670465 10.1097/j.pain.0000000000000454

[2] Global, regional, and national incidence, prevalence, and years lived with disability for 301 acute and chronic diseases and injuries in 188 countries, 1990-2013: a systematic analysis for the Global Burden of Disease Study 2013. Lancet 2015, 386:743–800. 26063472 10.1016/S0140-6736(15)60692-4 PMC4561509

[3] Blyth FM, Huckel Schneider C: Global burden of pain and global pain policy-creating a purposeful body of evidence. Pain 2018, 159:S43–S48. 30113946 10.1097/j.pain.0000000000001311

[4] Finnerup NB, : Pharmacotherapy for neuropathic pain in adults: a systematic review and meta-analysis. Lancet Neurol 2015, 14:162–173. 25575710 10.1016/S1474-4422(14)70251-0 PMC4493167

[5] Yekkirala AS, : Breaking barriers to novel analgesic drug development. Nat Rev Drug Discov 2017, 16:545–564. 28596533 10.1038/nrd.2017.87 PMC5675565

[6] Battaglia M, : We need to talk: the urgent conversation on chronic pain, mental health, prescribing patterns and the opioid crisis. J Psychopharmacol 2023, 37:437–448. 37171242 10.1177/02698811221144635

[7] Pain genes database. 09-16-2023]; Available from: https://www.re3data.org/repository/r3d100012129.

[8.**] Smith MT: Nonopioid analgesics discovery and the Valley of Death: EMA401 from concept to clinical trial. Pain 2022, 163:S15–S28. 35984369 10.1097/j.pain.0000000000002675 PMC10578428

[9] Eisenach JC, Rice ASC: Improving preclinical development of novel interventions to Treat pain: insanity is doing the same thing over and over and expecting different results. Anesth Analg 2022, 135:1128–1136. 36384008 10.1213/ANE.0000000000006249 PMC9976707

[10.*] Vieira TN, : Angiotensin type 2 receptor antagonism as a new target to manage gout. Inflammopharmacology 2022, 30:2399–2410. 36173505 10.1007/s10787-022-01076-x PMC7614762

[11] Shepherd AJ, : Angiotensin II triggers peripheral macrophage-to-sensory neuron redox crosstalk to elicit pain. J Neurosci 2018, 38:7032–7057. 29976627 10.1523/JNEUROSCI.3542-17.2018 PMC6083458

[12] Zhang H, : Structural basis for selectivity and diversity in angiotensin II receptors. Nature 2017, 544:327–332. 28379944 10.1038/nature22035 PMC5525545

[13] Perryman R, : Inhibition of the angiotensin II type 2 receptor AT2R is a novel therapeutic strategy for glioblastoma. Proc Natl Acad Sci USA 2022, 119, e2116289119. 35917342 10.1073/pnas.2116289119 PMC9371711

[14] Smith MT, Wyse BD, Edwards SR: Small molecule angiotensin II type 2 receptor (AT(2)R) antagonists as novel analgesics for neuropathic pain: comparative pharmacokinetics, radioligand binding, and efficacy in rats. Pain Med 2013, 14:692–705. 23489258 10.1111/pme.12063

[15] Smith MT, : A small molecule angiotensin II type 2 receptor (AT(2)R) antagonist produces analgesia in a rat model of neuropathic pain by inhibition of p38 mitogen-activated protein kinase (MAPK) and p44/p42 MAPK activation in the dorsal root ganglia. Pain Med 2013, 14:1557–1568. 23742186 10.1111/pme.12157

[16] Muralidharan A, Wyse BD, Smith MT: Analgesic efficacy and mode of action of a selective small molecule angiotensin II type 2 receptor antagonist in a rat model of prostate cancer-induced bone pain. Pain Med 2014, 15:93–110. 24433468 10.1111/pme.12258

[17] Anand U, : Mechanisms underlying clinical efficacy of Angiotensin II type 2 receptor (AT2R) antagonist EMA401 in neuropathic pain: clinical tissue and in vitro studies. Mol Pain 2015, 11:38. 26111701 10.1186/s12990-015-0038-x PMC4482278

[18] Shepherd AJ, Mohapatra DP: Attenuation of unevoked mechanical and cold pain hypersensitivities associated with experimental neuropathy in mice by angiotensin II type-2 receptor antagonism. Anesth Analg 2019, 128:e84–e87. 31094778 10.1213/ANE.0000000000003857 PMC6652216

[19] De Logu F, : Macrophages and Schwann cell TRPA1 mediate chronic allodynia in a mouse model of complex regional pain syndrome type I. Brain Behav Immun 2020, 88:535–546. 32315759 10.1016/j.bbi.2020.04.037

[20] De Logu F, : Schwann cells expressing nociceptive channel TRPA1 orchestrate ethanol-evoked neuropathic pain in mice. J Clin Invest 2019, 129:5424–5441. 31487269 10.1172/JCI128022 PMC6877331

[21] De Logu F, : Schwann cell TRPA1 mediates neuroinflammation that sustains macrophage-dependent neuropathic pain in mice. Nat Commun 2017, 8:1887 29192190 10.1038/s41467-017-01739-2 PMC5709495

[22] Macpherson LJ, : Noxious compounds activate TRPA1 ion channels through covalent modification of cysteines. Nature 2007, 445:541–545. 17237762 10.1038/nature05544

[23] Gylfadottir SS, : Analysis of macrophages and peptidergic fibers in the skin of patients with painful diabetic polyneuropathy. Neurology - Neuroimmunology Neuroinflammation 2022, 9:e1111. 34764216 10.1212/NXI.0000000000001111 PMC8587735

[24] Okamura A, : Upregulation of renin-angiotensin system during differentiation of monocytes to macrophages. J Hypertens 1999, 17:537–545. 10404956 10.1097/00004872-199917040-00012

[25] Shepherd AJ, : Macrophage angiotensin II type 2 receptor triggers neuropathic pain. Proc Natl Acad Sci U S A 2018, 115:E8057–E8066. 30082378 10.1073/pnas.1721815115 PMC6112686

[26] Koyama S, : An electroencephalography bioassay for preclinical testing of analgesic efficacy. Sci Rep 2018, 8,16402. 30401974 10.1038/s41598-018-34594-2 PMC6219560

[27.*] Guo Y, : Discovery and optimization of highly potent and selective AT(2)R antagonists to relieve peripheral neuropathic pain. ACS Omega 2021, 6:15412–15420. 34151119 10.1021/acsomega.1c01866 PMC8210434

[28] Murgasova R, : Non-clinical characterization of the disposition of EMA401, a novel small molecule angiotensin II type 2 receptor (AT2R) antagonist. Biopharm Drug Dispos 2020, 41:166–183. 32190910 10.1002/bdd.2226

[29] Smith MT, Anand P, Rice AS: Selective small molecule angiotensin II type 2 receptor antagonists for neuropathic pain: preclinical and clinical studies. Pain 2016, 157(Suppl 1):S33–S41. 26785154 10.1097/j.pain.0000000000000369

[30] Rice ASC, : EMA401, an orally administered highly selective angiotensin II type 2 receptor antagonist, as a novel treatment for postherpetic neuralgia: a randomised, double-blind, placebo-controlled phase 2 clinical trial. Lancet 2014, 383:1637–1647. 24507377 10.1016/S0140-6736(13)62337-5

[31.**] Rice ASC, : Efficacy and safety of EMA401 in peripheral neuropathic pain: results of 2 randomised, double-blind, phase 2 studies in patients with postherpetic neuralgia and painful diabetic neuropathy. Pain 2021, 162:2578–2589. 33675631 10.1097/j.pain.0000000000002252

[32] Baron R, : Peripheral neuropathic pain: a mechanism-related organizing principle based on sensory profiles. Pain 2017, 158:261–272. 27893485 10.1097/j.pain.0000000000000753 PMC5266425

[33] Demant DT, : The effect of oxcarbazepine in peripheral neuropathic pain depends on pain phenotype: a randomised, double-blind, placebo-controlled phenotype-stratified study. Pain 2014, 155:2263–2273. 25139589 10.1016/j.pain.2014.08.014

[34] Reimer M, : Sensory bedside testing: a simple stratification approach for sensory phenotyping. Pain Reports 2020, 5:e820. 32903958 10.1097/PR9.0000000000000820 PMC7447375

[35] Rice ASC, : Sensory profiling in animal models of neuropathic pain: a call for back-translation. Pain 2018, 159:819–824. 29300280 10.1097/j.pain.0000000000001138 PMC5911154

[36] Smith MT, : Analgesic efficacy of small-molecule angiotensin II type 2 receptor antagonists in a rat model of antiretroviral toxic polyneuropathy. Behav Pharmacol 2014, 25:137–146. 24525712 10.1097/FBP.0000000000000025

[37.*] Zanata GC, : Blockade of bradykinin receptors or angiotensin II type 2 receptor prevents paclitaxel-associated acute pain syndrome in mice. Eur J Pain 2021, 25:189–198. 32965065 10.1002/ejp.1660

[38] Chakrabarty A, Liao Z, Smith PG: Angiotensin II receptor type 2 activation is required for cutaneous sensory hyperinnervation and hypersensitivity in a rat hind paw model of inflammatory pain. J Pain 2013, 14:1053–1065. 23726047 10.1016/j.jpain.2013.04.002 PMC3971648

[39] Smith MT, Wyse BD: Method of treatment or prophylaxis. HK1103364A1. 2007 [Patent].

[40] Liao Z, : A local inflammatory renin-angiotensin system drives sensory axon sprouting in provoked vestibulodynia. J Pain 2017, 18:511–525. 28062309 10.1016/j.jpain.2016.12.008 PMC6261484

